# Supplementary material for: MAC-1 marks a quiescent and functionally superior HSC subset during regeneration
Source: Stem Cell Reports. 2023 Mar 2;18(3):736–48. doi: 10.1016/j.stemcr.2023.01.014 (PMC10031298; doi:10.1016/j.stemcr.2023.01.014)
Supplement: Document S1. Figures S1–S3 [file mmc1.pdf]

Stem Cell Reports, Volume *18*

Supplemental Information

MAC-1 marks a quiescent and functionally superior HSC subset during regeneration

Anna Rydström, Els Mansell, Valgardur Sigurdsson, Julia Sjöberg, Shamit Soneji, Kenichi Miharada, and Jonas Larsson

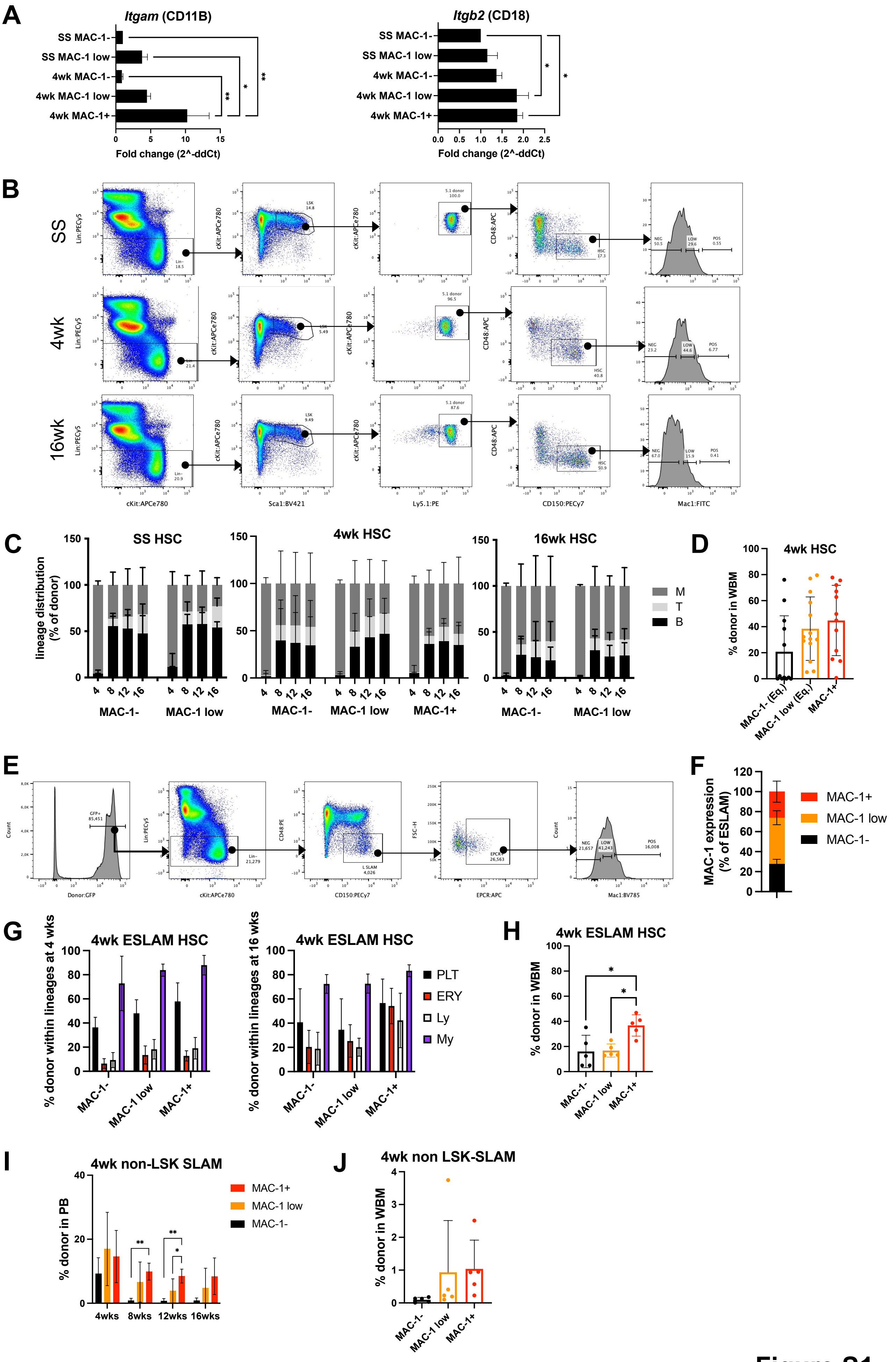
A
Itgam (CD11B)
SS MAC-1-
SS MAC-1 low
4wk MAC-1-
4wk MAC-1 low
4wk MAC-1+
Fold change (2^-ddCt)
Itgb2 (CD18)
B
SS
4wk
16wk
cKit:APCe780
Sca1:BV421
Ly5.1:PE
CD150:PECy7
Mac1:FITC
C
SS HSC
4wk HSC
16wk HSC
lineage distribution (% of donor)
MAC-1-
MAC-1 low
MAC-1+
M
T
B
D
4wk HSC
% donor in WBM
MAC-1- (Eq.)
MAC-1 low (Eq.)
MAC-1+
E
Donor:GFP
cKit:APCe780
CD150:PECy7
EPCR:APC
Mac1:BV785
F
MAC-1 expression (% of ESLAM)
MAC-1+
MAC-1 low
MAC-1-
G
4wk ESLAM HSC
% donor within lineages at 4 wks
% donor within lineages at 16 wks
PLT
ERY
Ly
My
H
4wk ESLAM HSC
% donor in WBM
I
4wk non-LSK SLAM
% donor in PB
4wks
8wks
12wks
16wks
J
4wk non LSK-SLAM
% donor in WBM

Figure S1

Figure S1. BM and PB analysis after transplantation. Related to Figure 1.
(A) Gene expression analysis by qPCR of *Itgam* and *Itgb2* in SS and 4wk HSCs sorted based on MAC-1 expression. Data are represented as mean ± SD. (n=3 mice). Statistical significance was determined using one-way ANOVA in combination with Turkey's multiple comparisons test (*p<0.05), (**p<0.01). (B) Representative dot plots of FACS of LSK SLAM HSCs from SS mice and from mice 4 or 16 weeks after transplantation. (C) Lineage output over time in donor PB shown in Figure 1D. Data are represented as mean ± SD. (D) Engraftment in BM 16 weeks after transplantation of equivalent cell numbers to 200 MAC-1^{+} HSCs. The MAC-1^{+} bar is the same as in (Figure 1E). Data pooled from 2 experiments (n=12-14 mice). Data are represented as mean ± SD. (E) Representative dot plots of FACS of ESLAM HSCs from mice 4 weeks after transplantation. (F) Proportion of HSCs in (E) being MAC-1^{-}, MAC-1^{low} and MAC-1^{+}. (G) Engraftment of 4wk ESLAM HSCs at 4 and 16 weeks in PB and at 16 weeks in BM (H). (n=5 mice for F-H). Data are represented as mean ± SD. Statistical significance was determined using one-way ANOVA in combination with Turkey's multiple comparisons test (*p<0.05), (**p<0.01). (I) Engraftment over time in donor PB and BM (J) after transplantation of 4wk non-LSK SLAM cells. (n=5 mice). Data are represented as mean ± SD. Statistical significance was determined using one-way ANOVA in combination with Turkey's multiple comparisons test (*p<0.05), (**p<0.01).

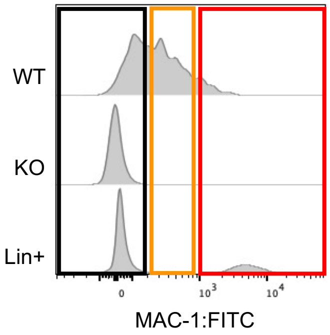

Figure S2

Figure S2. MAC-1 expression in WT and *Itgam* KO HSCs. Related to Figure 2.
Histograms of HSCs (LSK-SLAM) from WT and KO mice 4 weeks after transplantation. Lineage positive (Lin^+) cells are displayed as reference.

A

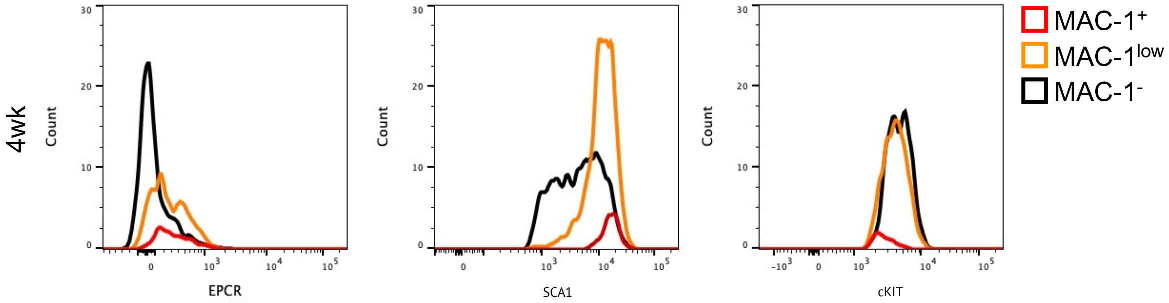

B

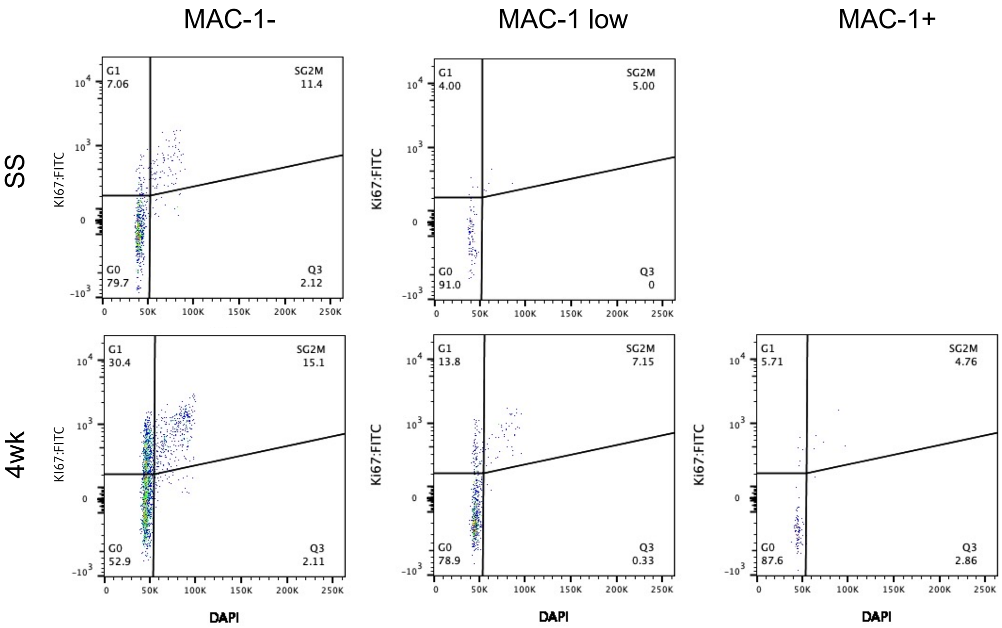

C

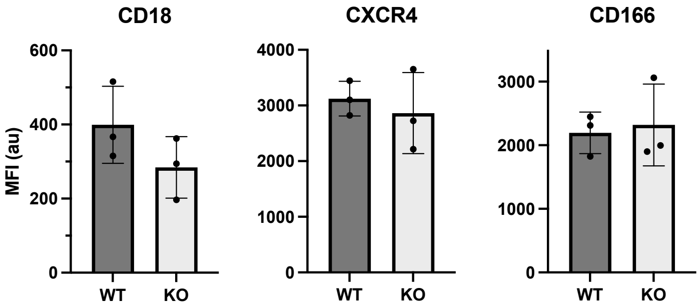

Figure S3

Figure S3. Protein expression and cell cycle analysis on HSCs. Related to Figure 3.
(A) Representative histograms of cell surface protein expression of EPCR, SCA1 and cKIT on 4wk MAC-1 LSK SLAM HSC subpopulations. (B) Representative dot plots of flow cytometry analysis of HSCs from SS mice and from mice 4 weeks after transplantation based on their MAC-1 expression. (C) Protein expression of adhesion molecules CD18, CXCR4 and CD166 on HSCs from *Itgam* KO and WT littermates. (n=3 mice). Data are represented as mean ± SD.
