# Supplementary material for: MAC-1 marks a quiescent and functionally superior HSC subset during regeneration
Source: Stem Cell Reports. 2023 Mar 2;18(3):736–48. doi: 10.1016/j.stemcr.2023.01.014 (PMC10031298; doi:10.1016/j.stemcr.2023.01.014)
Supplement: Document S2. Article plus supplemental information [file mmc2.pdf]

Stem Cell Reports
Article
OPEN ACCESS

MAC-1 marks a quiescent and functionally superior HSC subset during regeneration

Anna Rydström,[1] Els Mansell,[1,2] Valgardur Sigurdsson,[1] Julia Sjöberg,[1] Shamit Soneji,[3] Kenichi Miharada,[4] and Jonas Larsson[1,*]

[1]Molecular Medicine and Gene Therapy, Lund Stem Cell Center, Lund University, BMC A12, 221 84 Lund, Sweden
[2]Stem Cell Group, Cancer Institute, University College London, London, UK
[3]Division of Molecular Hematology, Lund Stem Cell Center, Lund University, Lund, Sweden
[4]International Research Center for Medical Sciences, Kumamoto University, Kumamoto, Japan
*Correspondence: jonas.larsson@med.lu.se
https://doi.org/10.1016/j.stemcr.2023.01.014

SUMMARY

Mouse hematopoietic stem cells (HSCs) have been extensively defined both molecularly and functionally at steady state, while regenerative stress induces immunophenotypical changes that limit high purity isolation and analysis. It is therefore important to identify markers that specifically label activated HSCs to gain further knowledge about their molecular and functional properties. Here, we assessed the expression of macrophage-1 antigen (MAC-1) on HSCs during regeneration following transplantation and observed a transient increase in MAC-1 expression during the early reconstitution phase. Serial transplantation experiments demonstrated that reconstitution potential was highly enriched in the MAC-1$^+$ portion of the HSC pool. Moreover, in contrast to previous reports, we found that MAC-1 expression inversely correlates with cell cycling, and global transcriptome analysis showed that regenerating MAC-1$^+$ HSCs share molecular features with stem cells with low mitotic history. Taken together, our results suggest that MAC-1 expression marks predominantly quiescent and functionally superior HSCs during early regeneration.

INTRODUCTION

Bone marrow transplantation has been used in the clinic since the 1950s and has become a common treatment for a number of blood disorders. Bone marrow transplantation has the potential to give a lifelong cure owing to the properties of the hematopoietic stem cells (HSCs) to both self-renew and differentiate into all blood lineages. Following transplantation, HSCs undergo extensive proliferation to meet the demand of replenishing the hematopoietic system. This is in stark contrast to the homeostatic context, where the vast majority of HSCs reside in a quiescent state.

Proliferation and differentiation of HSCs must be tightly regulated during conditions of stress and acute regeneration to allow for replenishment of mature blood cells and at the same time prevent exhaustion of the stem cell pool. However, the programs regulating stemness and differentiation are dysregulated in many leukemias. In this context, quiescence is key to protecting HSCs from stress-induced insults that may predispose to hematological malignancies (Bowman et al., 2018; Walter et al., 2015). It is a long-standing goal in the field to better understand the mechanisms regulating HSC activation, but a key challenge in this regard is to isolate activated HSCs at high purity. While steady-state murine HSCs can be efficiently purified using specific combinations of cell surface markers (Lin$^-$, SCA1$^+$, cKIT$^+$, CD48$^-$, CD150$^+$ [LSK-SLAM]) (Kiel et al., 2005), some of these markers display an altered expression during severe stress, making it challenging to isolate actively regenerating HSCs (Randall and Weissman, 1997).

Macrophage-1 antigen (MAC-1) is a cell surface marker typically expressed on mature macrophages and natural killer (NK) cells. MAC-1 is a heterodimer consisting of CD11B and CD18 and mediates a number of cellular responses such as migration, adhesion, and phagocytosis. MAC-1 is normally absent or expressed at very low levels in HSCs and is therefore often used as a negative selection marker. However, it has been shown that the cytotoxic drug 5-fluorouracil (5FU) activates HSCs and triggers an upregulation of MAC-1 such that functional HSCs are found exclusively within the MAC-1^{low} fraction of 5FU-treated mice (Randall and Weissman, 1997; Wang et al., 2019). This report further showed that 25% of hematopoietic stem and progenitor cells (LSK) in 5FU-treated mice are undergoing cycling. Furthermore, fetal liver HSCs that are highly proliferative have been shown to express MAC-1 (Morrison et al., 1995). Altogether, these findings have led to the assumption that MAC-1 expression correlates with an active cell-cycle state. Yet, it remains unclear to date if MAC-1 has a functional role in HSC activation. While mouse knockout models for both of the two MAC-1 subunits have been established and shown to have perturbed neutrophil functionality, an extensive analysis of the hematopoietic stem and progenitor cell (HSPC) compartment has not been performed in either of these models (Coxon et al., 1996; Scharffetter-Kochanek et al., 1998). More recent studies of both mouse and human

This is an open access article under the CC BY license (http://creativecommons.org/licenses/by/4.0/).

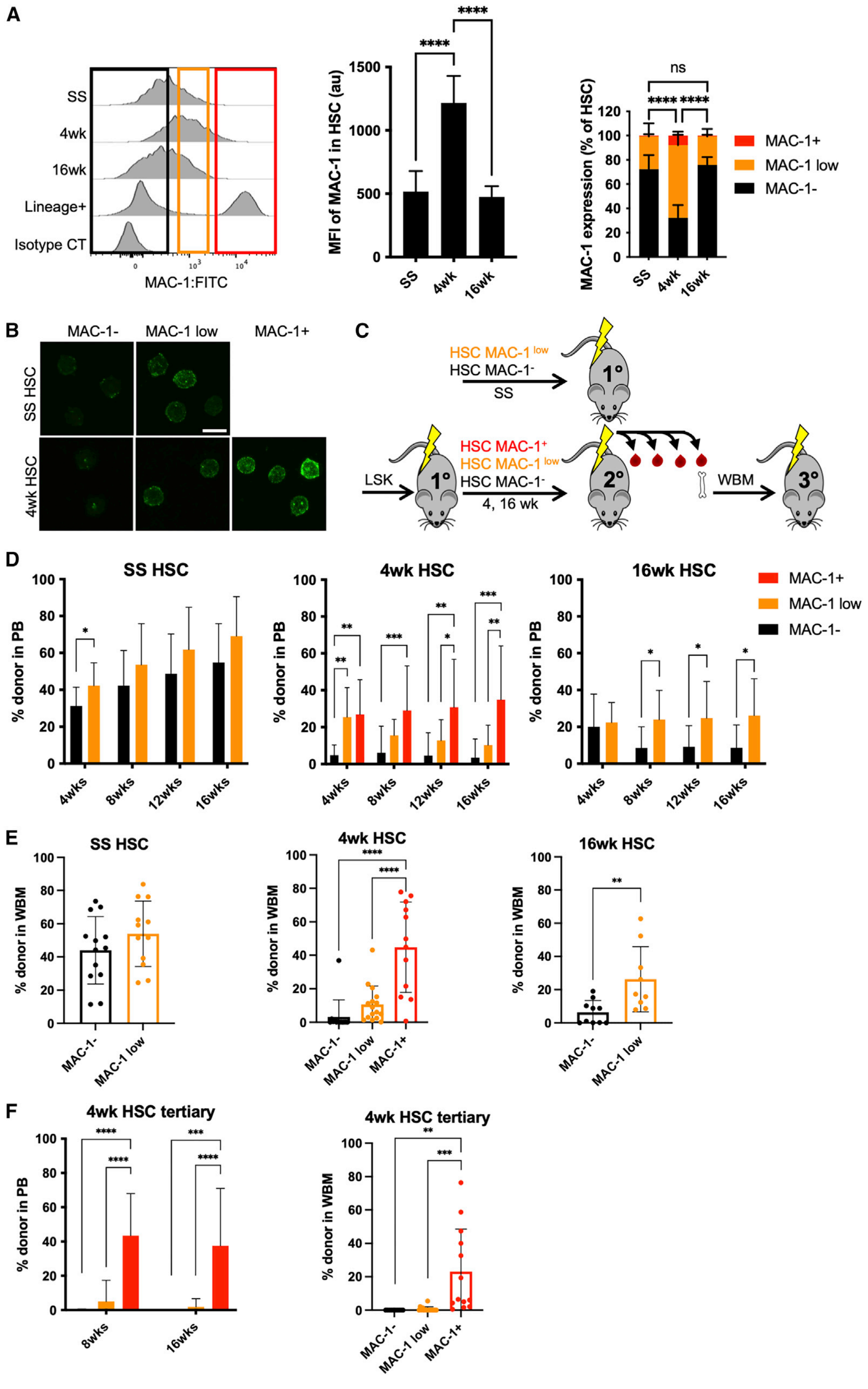

(legend on next page)

HSPCs with impaired MAC-1 signaling showed compromised engraftment, suggesting that MAC-1 could be implicated in the interaction between HSCs and the bone marrow niche after transplantation (Mei et al., 2020; Prashad et al., 2015).

Here, we have further investigated the role of MAC-1 in HSCs during regenerative stress after transplantation. We show that there is a transient increase in MAC-1 expression during the early regenerative phase that positively correlates with both reconstitution potential and cellular quiescence, as well as a gene expression profile indicative of low mitotic history.

Importantly, our findings allow for the identification of a small subpopulation of highly purified long-term HSCs during regeneration.

RESULTS

MAC-1 is upregulated upon transplantation and marks HSCs with high reconstitution potential

To assess expression levels of MAC-1 in LSK-SLAM HSCs during hematopoietic regeneration, we analyzed bone marrow 4 and 16 weeks after transplantation to recapitulate both the early and late phases of regeneration. Flow cytometry analysis showed that HSCs isolated 4 weeks after transplantation (4 week HSCs) had higher expression of MAC-1 compared with both non-transplanted steady-state HSCs (SS HSCs) and HSCs isolated 16 weeks after transplantation (16 week HSCs) (Figure 1A). The majority (70%) of both SS and 16 week HSCs completely lacked MAC-1 expression (MAC-1^{-}), with the remaining fraction showing low levels of expression (MAC-1^{low}). By contrast, the majority of 4 week HSCs were MAC-1^{low}, with a small but distinct population of HSCs with high MAC-1 expression (MAC-1^{+}) found exclusively in 4 week HSCs. We confirmed the observed protein expression pattern of MAC-1 by fluorescence microscopy (Figure 1B), and this correlated with the mRNA expression levels validated by qPCR (Figure S1A). Next, we wanted to functionally assess these HSC subpopulations with varying levels of MAC-1 expression through serial transplantation (Figures 1C and S1B). Upon transplantation, MAC-1^{-} and MAC-1^{low} SS HSCs showed comparable reconstitution potential and output in peripheral blood over time, as well as in bone marrow (Figures 1D and 1E). MAC-1^{low} HSCs isolated 16 weeks after primary transplantation performed better than MAC-1^{-} HSCs. Most strikingly, we found that the discrete fraction of MAC-1^{+} HSCs isolated during the early regenerative phase (4 week HSCs) showed significantly higher long-term reconstitution compared with both the MAC-1^{low} and MAC-1^{-} 4 week HSCs in both the peripheral blood and bone marrow. Moreover, upon tertiary bone marrow transplantation, we could exclusively detect robust serial engraftment capacity from the MAC-1^{+} group isolated 4 weeks after primary transplantation but not from mice initially transplanted with MAC-1^{low} or MAC-1^{-} HSCs (Figure 1F). These results indicate that the most potent HSCs during early regeneration express MAC-1. We did not observe any significant differences in blood lineage output between the MAC-1^{+}, MAC-1^{low}, and MAC-1^{-} populations from any given time point (Figure S1C). We did, however, observe a general myeloid bias acquired with transplantation history as well as chronological age.

While these experiments show that the small MAC-1^{+} subset of 4 week HSCs was highly enriched for functional repopulating cells, the difference in size of the MAC-1 subpopulations prompted us to also assess the total content of functional HSC activity within each population.

Figure 1. Transient MAC-1 expression after transplantation reflects reconstitution potential in HSCs

(A) MAC-1 expression in LSK SLAM HSCs at steady state (SS) or after 4 and 16 weeks of transplantation analyzed by flow cytometry. MFI (middle) and proportion of HSCs being MAC-1^{-}, MAC-1^{low}, and MAC-1^{+} (right). Asterisks denote all three MAC-1 populations to be significantly different at 4 weeks compared with both SS and 16 week HSCs (SS MAC-1^{-} vs. 4 week MAC-1^{-} $p < 0.0001$; 4 week MAC-1^{-} vs. 16 week MAC-1^{-} $p < 0.0001$; SS MAC-1^{low} vs. 4 week MAC-1^{low} $p < 0.0001$; 4 week MAC-1^{low} vs. 16 week MAC-1^{low} $p < 0.0001$; SS MAC-1^{+} vs. 4 week MAC-1^{+} $p < 0.0001$; 4 week MAC-1^{+} vs. 16 week MAC-1^{+} $p < 0.0001$). Data pooled from 2 experiments (n = 6–16 mice).

(B) MAC-1 expression in SS and 4 week HSCs analyzed by fluorescence microscopy. Scale bar, 10 μm.

(C) SS HSCs were sorted based on MAC-1 expression and transplanted to irradiated primary recipients (200 HSCs/recipient). LSK cells were sorted and transplanted to irradiated primary recipients. 4 and 16 weeks after primary transplantation, HSCs were sorted based on MAC-1 expression and transplanted to irradiated secondary recipients (200 HSCs/recipient). WBM from secondary recipients initially transplanted with 4 week HSCs were transplanted to irradiated tertiary recipients. Data pooled from 2 experiments (n = 9–16 mice).

(D) Engraftment over time in peripheral blood (PB) from experiment described in (C).

(E) Engraftment in bone marrow (BM) after 16 weeks from experiment described in (C).

(F) Engraftment in PB and BM of mice receiving a serial WBM transplantation from mice initially transplanted with 4 week HSCs. Data pooled from 2 experiments (n = 10–16 mice). Data are represented as mean ± SD. Statistical significance was determined using two-tailed unpaired Student's t test or one-way ANOVA in combination with Turkey's multiple comparisons test (*$p < 0.05$, **$p < 0.01$, ***$p < 0.001$, and ****$p<0.0001$).

See also Figure S1.

To this end, we transplanted the equivalent number to 200 MAC-1$^+$ HSCs (i.e., 1,500 MAC-1^{low} cells and 800 MAC-1$^-$ cells) and measured long-term engraftment in bone marrow (Figure S1D). Although the assay was not done at limit dilution and therefore is not quantitative, the engraftment levels from these equivalent cell doses indicate a higher functional HSC content in the MAC-1$^+$ and MAC-1^{low} fractions compared with the MAC-1$^-$ population.

SCA1 and cKIT have been reported to exhibit an altered expression upon inflammation and proliferative stress (Pietras et al., 2014; Randall and Weissman, 1997), which is why we also wanted to explore the use of MAC-1 by applying an alternative HSC definition. We therefore transplanted LSK cells from GFP donor mice and analyzed MAC-1 expression in GFP$^+$, Lin$^-$, CD48$^-$, CD150$^+$, EPCR$^+$ (ESLAM) HSCs (Figures S1E and S1F). Similar to 4 week LSK SLAM HSCs, the 4 week ESLAM population contained MAC-1$^-$, MAC-1^{low}, and MAC-1$^+$ cells. When transplanted, the MAC-1$^+$ ESLAM HSCs displayed a trend of higher reconstitution in all blood lineages, and a significantly higher engraftment in the bone marrow, compared with MAC-1$^-$ and MAC-1^{low} ESLAM HSCs, suggesting that upregulation of MAC-1 is a general feature of regenerating HSCs, irrespective of the phenotypic definition (Figures S1G and S1H).

Finally, we investigated the possibility of MAC-1 upregulation on any HSCs residing outside the classical LSK SLAM gate during regeneration. We sorted 4 week non-LSK SLAM cells based on MAC-1 expression and transplanted the equivalent dose of each population, to that of 200 MAC-1$^+$ LSK SLAM HSCs. As expected, reconstitution levels in blood and bone marrow were overall very low, but there was a trend of higher engraftment from MAC-1$^+$ non-LSK SLAM cells, suggesting that MAC-1 marks HSCs outside the conventional HSC definition during regeneration (Figures S1I and S1J).

Taken together, our findings demonstrate that MAC-1 is upregulated during early regeneration and uniquely marks a small population of the most functionally potent HSCs.

MAC-1 is dispensable for the regenerative capacity of HSCs

Next, we set out to investigate if the observed upregulation of MAC-1 upon regenerative stress plays a direct role in HSC behavior and function. To this end, we used a conventional knockout mouse model of the CD11B subunit (*Itgam*) where homozygous mice lack expression of MAC-1 (Coxon et al., 1996). We first characterized the stem and progenitor cells in the bone marrow of SS mice and observed no difference in the frequencies of HSCs and various progenitor populations between knockout (KO) and wild-type (WT) littermates (Figures 2A and 2B). Additionally, we found that the cell-cycle state of HSCs was unaffected by the lack of MAC-1 expression (Figure 2C). Next, to assess the role of MAC-1 during active regeneration, we performed a competitive primary transplantation of WT or littermate *Itgam* KO LSK cells (Ly 5.2) together with equal numbers of competitor cells (LY5.1) (Figure 2D). Following 4 weeks of engraftment, we sorted HSCs from the competing populations, and we confirmed the absence of MAC-1 expression on KO HSCs and upregulation of MAC-1 on transplanted WT HSCs (Figure S2). Now, to interrogate the correlation between MAC-1 expression and functional HSC activity, these HSCs were re-transplanted in a competitive setting (1:1 ratio) into secondary recipients. Analysis of the primary and secondary recipients showed largely comparable engraftment levels and HSPC distribution between WT and KO cells (Figures 2E–2J), indicating that the functional reconstitution potential of HSCs remains intact in the absence of MAC-1 both at SS and during active regeneration. In the secondary recipients, we observed a small but significant increase in B lineage output as well as a moderate increase in overall donor contribution in bone marrow from the KO cells (Figures 2H–2J). Our findings therefore suggest that MAC-1 is dispensable for the regenerative capacity of HSC and that its functional involvement is not implicated in the competitive advantage of regenerating MAC-1$^+$ HSCs.

MAC-1 marks predominantly quiescent HSCs with distinct transcriptional features

To identify molecular differences underlying the functional heterogeneity of HSCs that differ in MAC-1 expression, we performed RNA sequencing on the SS and 4 week HSC subpopulations (SS MAC-1$^-$, SS MAC-1^{low}, 4 week MAC-1$^-$, 4 week MAC-1^{low}, and 4 week MAC-1$^+$). Principal-component analysis (PCA) of the 1,000 most variable genes separated all the populations accordingly (Figure 3A). In particular, MAC-1 expression could efficiently separate three transcriptionally distinct populations of early regenerating HSCs corresponding to their differential regenerative capacity upon transplantation.

Of the 3,452 genes differentially expressed between the five HSC subpopulations (log2 fold change [FC] > 1, adjusted p value [padj] < 0.1), we identified several HSC signature genes that were stably expressed in SS HSCs and that progressively increased with elevated MAC-1 expression in the 4 week HSCs (*Mpl*, *Fgd5*, *Uba7*, *Procr*, *Glipr1l1*, *Nupr1*, *Hlf*, *Mecom*, *Socs2*, *Mllt3*, *Ly6a*, *Kit*), in line with the functional difference observed between 4 week MAC-1$^-$ and 4 week MAC-1$^+$ cells (Figure 3B). A similar trend was observed at the protein level for *Procr* (EPCR) and *Ly6a* (SCA1), while *Kit* showed an inverse correlation between mRNA and protein expression (Figure S3A). Furthermore, 4 week MAC-1$^-$ HSCs expressed high levels of genes

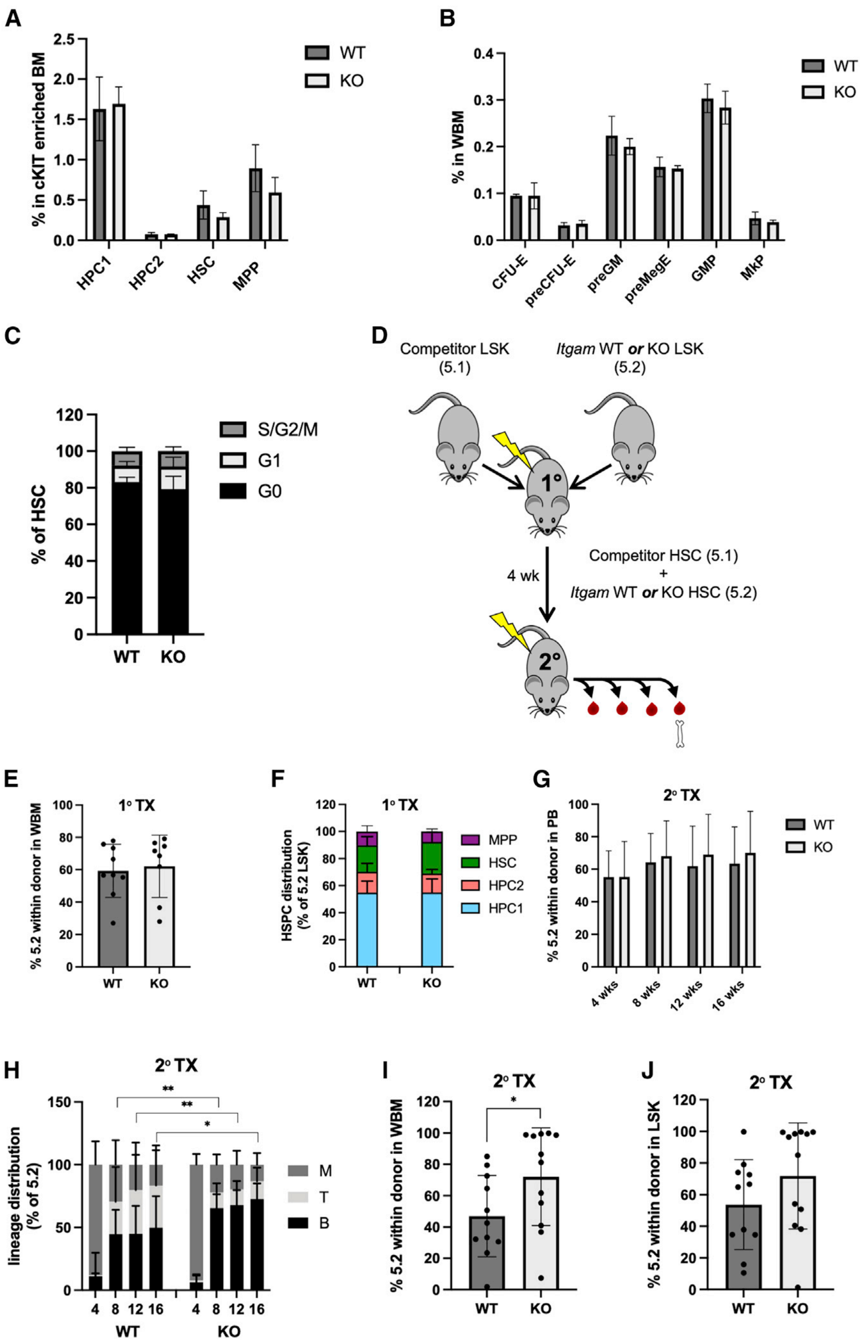

Figure 2. MAC-1 expression is not required for HSC function during homeostasis or regeneration

(A) Frequency of stem and progenitor populations in cKIT-enriched BM of WT and *Itgam*-KO SS mice. HPC1 (LSK, $CD48^{+}$, $CD150^{-}$); HPC2 (LSK, $CD48^{+}$, $CD150^{+}$); MPP (LSK, $CD48^{-}$, $CD150^{-}$) (n = 3 mice).

(B) Frequency of myeloerythroid progenitor populations in WBM of WT and KO SS mice (n = 3 mice).

(C) Cell-cycle analysis of WT and KO HSCs at SS. Data pooled from 2 experiments (n = 6 mice).

(D) Equal number or competitor (LY5.1) and *Itgam* WT or KO (LY5.2) LSK cells were transplanted to lethally irradiated primary recipients. After 4 weeks, equal numbers of competitor (LY5.1) and *Itgam* WT or KO (LY5.2) HSCs were sorted and transplanted to lethally irradiated secondary recipients.

(E) LY5.2 donor engraftment in WBM in primary recipients after 4 weeks. Data pooled from 2 experiments (n = 8–9 mice).

(F) Stem and progenitor distribution within LSK from BM analyzed in (E).

(G) LY5.2 donor engraftment over time in PB in secondary recipients. Data pooled from 2 experiments (n = 11–12 mice).

(H) Lineage output in LY5.2 PB over time in secondary recipients.

(I) LY5.2 donor engraftment in WBM in secondary recipients.

(J) LY5.2 donor engraftment in LSK in secondary recipients. Engraftment was calculated as % LY5.2 within the donor derived cells. Data are represented as mean ± SD. Statistical significance was determined using two-tailed unpaired Student's t test ($^{*}p < 0.05$ and $^{**}p<0.01$).

See also Figure S2.

involved in megakaryocytic/erythroid lineage commitment (*Gata1*, *Mfsd2b*, *Klf1*, *Vwf*, *Itga2b*, *Pf4*, *Gp9*, *Gfi1b*) and low levels of the lymphoid and myeloid priming genes (*Flt3*, *Ets*, *Ikzf1*, *Notch2*, *CD86*, *Spi1*, *Gfi1*) (Figure 3B).

The striking difference observed between 4 week MAC-1^{-} and MAC-1^{+} HSCs prompted us to further characterize these populations using an unbiased global approach. We therefore performed gene set enrichment analysis (GSEA) and found an enrichment for genes involved in MYC signaling, cell cycle, and oxidative phosphorylation in the MAC-1^{-} HSCs (Figure 3C). These are processes previously described to impair HSC function and therefore provide molecular support to our observation that MAC-1^{-} HSCs show lower engraftment capacity. MAC-1^{-} HSCs were also enriched for genes involved in megakaryocyte differentiation, while MAC-1^{+} HSCs were enriched for genes linked to myeloid and lymphoid differentiation. Although our *in vivo* analyses of the MAC-1

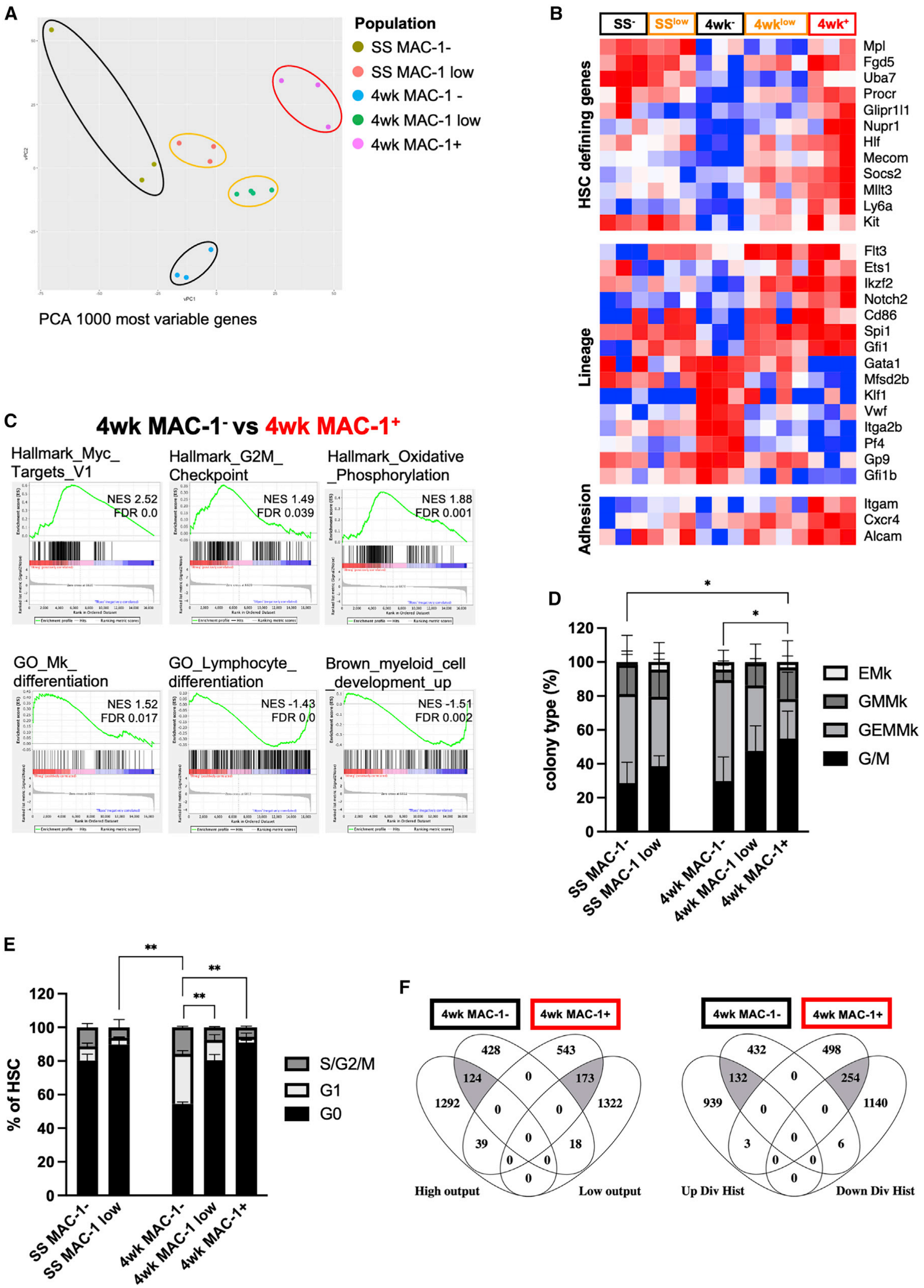

(legend on next page)

subpopulations did not indicate a lineage bias, we wanted to investigate if these transcriptional signatures would reflect their immediate potential to produce megakaryocytes, erythrocytes, and myeloid cells *in vitro* by performing a colony-forming unit (CFU) analysis. We sorted cells from SS and 4 week mice and observed a small but significant increase in granulocyte/monocyte (GM) and a decrease in granulocyte/erythrocyte/monocyte/megakaryocyte (GEMMk) colonies formed from 4 week MAC-1^{+} HSCs (Figure 3D), suggesting that MAC-1^{+} cells have a preference to produce myeloid cells over erythroid or megakaryocytic cells *in vitro*. The more balanced output observed *in vivo* may indicate a functional difference in the long-term vs. immediate production of progeny inherent to these HSC subpopulations.

Our GSEA analysis had indicated that MAC-1^{+} regenerating HSCs are less cycling compared with their MAC-1^{-} counterparts. To confirm this, we performed cell-cycle analysis, which indeed revealed that MAC-1^{+} early regenerating HSCs are significantly more quiescent than MAC-1^{-} HSCs (Figures 3E and S3B). Importantly, this contrasts with the current notion that MAC-1 expression in HSCs is associated with increased cell cycling (Randall and Weissman, 1997). An important aspect of HSC function is the ability to transition between an activated state and quiescence. It has previously been shown that transplantation into irradiated recipients recruits the entire stem cell population into extensive proliferation (Takizawa et al., 2011). The less-cycling MAC-1^{+} population may thus represent the first HSCs to return to quiescence following regenerative stress. In line with this, we found that *Socs2* and *Hlf*, both regulators of HSC quiescence during stress (Komorowska et al., 2017; Vitali et al., 2015), are expressed at higher levels in 4 week MAC-1^{+} HSCs (Figure 3B). Moreover, the adhesion molecules *Cxcr4*, *Alcam*, and *Notch2*, which are important for anchoring HSCs in their niche (Chitteti et al., 2014; Foudi et al., 2006; Jeannet et al., 2013; Nie et al., 2008; Wang et al., 2015), were all expressed at higher levels in 4 week MAC-1^{+} HSCs compared with both 4 week MAC-1^{-} and SS HSCs (Figure 3B). To explore the possibility of a compensatory expression of other adhesion molecules than MAC-1 in the *Itgam* KO mouse, we analyzed the expression of CD18, CXCR4, and CD166 (*Alcam*) in WT and KO littermates but did not observe any significant difference in the expression of these proteins (Figure S3C).

It has been demonstrated that HSC functionality is strongly linked to divisional history where the number of divisions negatively correlates with long-term reconstitution potential (Qiu et al., 2014; Wilson et al., 2008). We compared the differentially expressed genes between 4 week MAC-1^{+} and MAC-1^{-} HSCs with datasets of HSCs with a different historic contribution to mature blood (Rodriguez-Fraticelli et al., 2020) or with a different divisional history (Bernitz et al., 2020). We found a specific and significant overlap between genes upregulated in 4 week MAC-1^{+} HSCs and genes upregulated in HSCs with low progeny output (representation factor 3.8; $p < 0.001$) as well as with genes downregulated with divisional history (representation factor 6; $p < 0.001$). Inversely, genes upregulated in 4 week MAC-1^{-} HSCs overlapped with genes upregulated in HSCs with high progeny output (representation factor 3.7; $p < 0.001$) and high divisional history (representation factor 5.4; $p < 0.001$) (shaded sectors in Figure 3F). Of note, the gene sets representing divisional history had been depleted of cell-cycle-related genes, suggesting that the molecular similarity with 4 week MAC-1^{-} HSCs goes beyond the cell-cycle state. In conclusion, our transcriptional analysis shows that MAC-1 marks a population of more quiescent HSCs and indicates that MAC-1 expression in regenerating HSCs may correlate with mitotic history.

MAC-1 expression delimits functionally distinct HSC subsets following 5-FU treatment

Finally, we sought to investigate if the correlation between MAC-1 expression, cell-cycle status, and HSC function was

Figure 3. Bulk RNA-seq analysis of SS and 4 week HSCs sorted based on MAC-1 expression

(A) Principal-component analysis (PCA) of SS MAC-1^{-}, SS MAC-1^{low}, 4 week MAC-1^{-}, 4 week MAC-1^{low}, and 4 week MAC-1^{+} HSCs. Output of the 1,000 most variable genes (n = 3–4 for Figures 4A–4C and 4F).

(B) Heatmap of differentially expressed genes important for HSC identity, lineage differentiation, and adhesion (log2FC > 1, padj < 0.1).

(C) Gene set enrichment analysis (GSEA) of 4 week MAC-1^{-} vs. 4 week MAC-1^{+} HSCs.

(D) Methylcellulose colony assay of HSCs at SS and 4 weeks after transplantation. Colonies were analyzed using flow cytometry. Data pooled from 2 experiments (n = 4–7 mice). Data are represented as mean ± SD. Statistical significance was determined using one-way ANOVA in combination with Turkey's multiple comparisons test (*p < 0.05 and **p < 0.01).

(E) Cell cycle analysis of HSCs at SS and 4weeks after transplantation based on MAC-1 expression. (n = 3 mice). Data are represented as mean ± SD. Statistical significance was determined using one-way ANOVA in combination with Turkey's multiple comparisons test (*p < 0.05 and **p < 0.01).

(F) Venn diagram showing overlap of differentially expressed genes between MAC-1^{+} and MAC-1^{-} 4 week HSC genes and published gene lists of historic low or high output (left) and divisional history (right).

See also Figure S3.

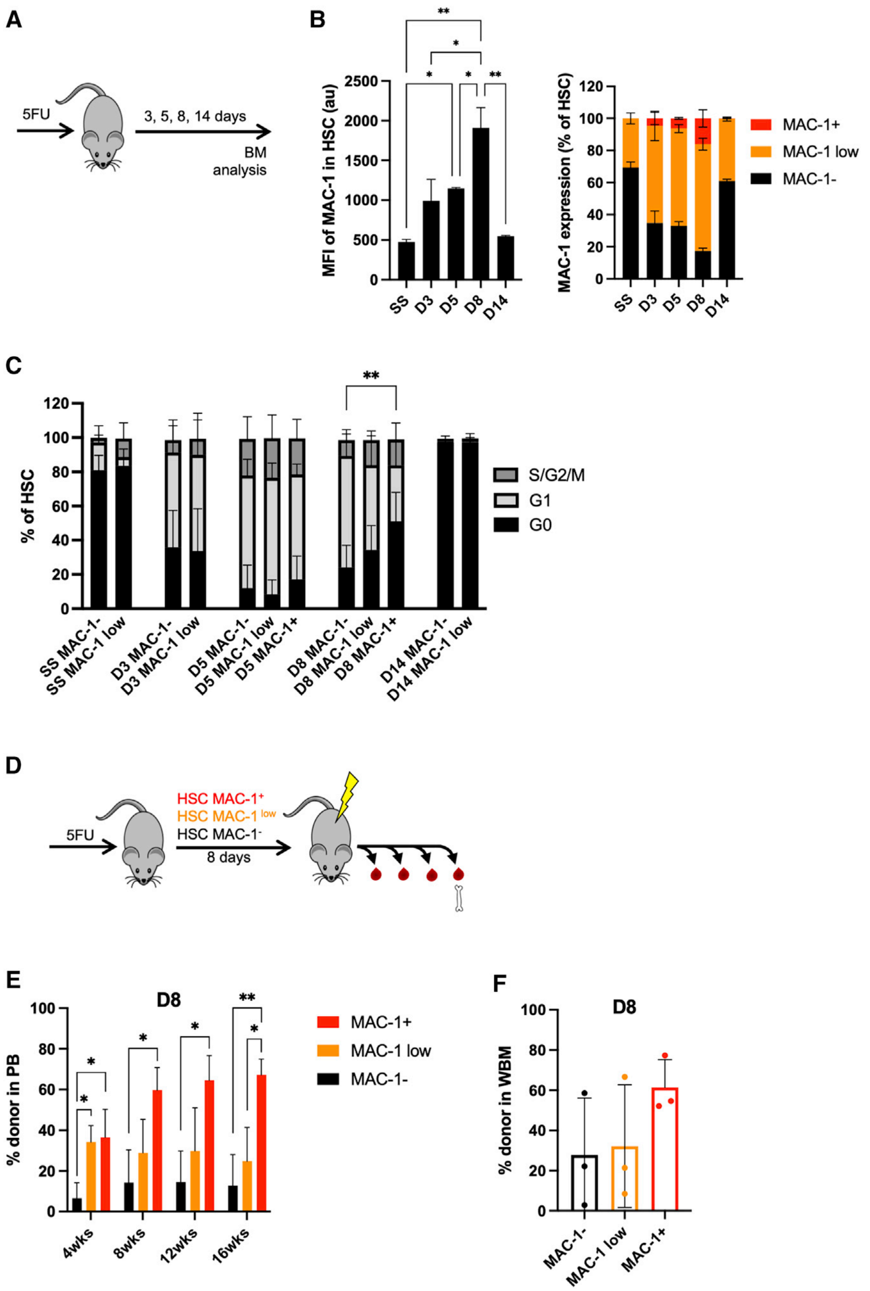

Figure 4. MAC-1 expression during recovery phase after 5FU

(A) Experimental setup of BM analysis after 5FU intravenous (i.v.) injections.

(B) MAC-1 expression in LS-SLAM HSCs after 5FU treatment. Mean fluorescence intensity (MFI; left) and proportion of HSCs being MAC-1^{-}, MAC-1^{low}, and MAC-1^{+} (right) (n = 2–4 mice).

(C) Cell-cycle analysis of SS HSCs and HSCs 3, 5, 8, and 14 days after 5FU. Data pooled from 2 experiments (n = 3–7 mice).

(D) 8 days after 5FU, HSCs were sorted based on MAC-1 expression and transplanted to irradiated recipients (200 HSCs/recipient).

(E and F) Engraftment over time in PB (E) and BM (F) from experiment described in (D) (n = 3 mice). Data are represented as mean ± SD. Statistical significance was determined using one-way ANOVA in combination with Turkey's multiple comparisons test (*$p < 0.05$ and **$p < 0.01$).

specific to regeneration post-transplantation or a general feature in response to severe stress. To this end, we analyzed MAC-1 expression in LS-SLAM HSCs at various time points after 5FU treatment, a chemotherapeutic agent known to cause myeloablation and rapid HSC activation. We observed that MAC-1 expression peaks 8 days after 5FU, with levels normalizing back to SS levels at day 14 (Figures 4A and 4B). Similar to our observations after transplantation, we could identify a small but significant transient population of MAC-1^{+} HSCs after 5FU treatment. When investigating how cell cycle correlated with the expression of MAC-1, we found that the vast majority of all MAC-1 HSC subsets were actively cycling after 5 days (Figure 4C). Strikingly, however, at day 8 following 5FU treatment, we found that the MAC-1^{+} HSCs displayed a significantly higher fraction of cells in G0, indicating that this subset had a propensity for a quicker return to a quiescent state.

Finally, to assess whether MAC-1 expression delimits functionally different HSC subsets also in the context of 5FU exposure, we sorted and transplanted 200 HSCs based on their MAC-1 expression 8 days following 5FU treatment

(Figure 4D). Again, MAC-1$^+$ HSCs had significantly higher long-term reconstitution in peripheral blood compared with MAC-1^{low} and MAC-1$^-$ HSCs (Figure 4E). Analysis of the bone marrow after 16 weeks displayed a similar trend (Figure 4F). We conclude that MAC-1 expression is highest in a small subset of more quiescent HSCs following severe stress, thereby demarcating cells with a distinct functional advantage over cells with lower MAC-1 expression.

DISCUSSION

In this article, we present evidence that MAC-1 upregulation is a common feature in HSCs as a response to severe mitotic stress. 5FU treatment and transplantation after radiation conditioning induce cycling of a vast majority of or the entire HSC population (Takizawa et al., 2011; Wilson et al., 2008). We demonstrate that both these stressors induce a transient increase of MAC-1 expression in HSCs. From a practical standpoint, this emphasizes the importance of not excluding MAC-1-expressing cells when staining for HSCs using lineage markers in combination with HSC-specific markers or during a lineage-depletion enrichment step.

Conventionally, increased MAC-1 expression has been thought to reflect activated and the most rapidly cycling HSCs. In contrast to this notion, our findings may suggest that HSCs with the highest MAC-1 expression constitute a small subpopulation that has returned to quiescence after extensive proliferation. Not only were regenerating MAC-1$^+$ HSCs more quiescent than their MAC-1$^-$ counterparts, but our transcriptomic analysis also revealed that genes upregulated in MAC-1$^+$ cells were overlapping with gene sets both from HSCs with low historic progeny output and with low divisional history. Although not formally demonstrated by our data, it is tempting to speculate that the MAC-1$^-$ HSCs have been the main contributors to the initial phase of hematopoietic regeneration, and thereby are the first to become exhausted, while MAC-1 is expressed by HSCs that primarily have divided symmetrically to expand the stem cell pool. These HSCs with low mitotic history may upregulate a battery of adhesion molecules, including MAC-1, thereby enhancing their homing ability and facilitating interactions with specific niches in the bone marrow that favor quiescence and preserve HSC function.

Using a conventional KO mouse model, we here demonstrate that the increase in expression of MAC-1 is not required per se for efficient HSC engraftment and regeneration. This does not exclude, however, that it acts together with other adhesion molecules with partially overlapping functions. Indeed, we detected increased levels of *Notch2*, *Cxcr4*, and *Alcam*, all with described functions in HSC niche interaction and quiescence (Chitteti et al., 2014; Foudi et al., 2006), in cells with the highest MAC-1 expression after transplantation. Interestingly, the expression of these adhesion molecules was higher in 4 week MAC-1$^+$ HSCs also compared with SS HSCs. This could be due to alterations in the niche itself as a result of radiation damage (Cao et al., 2011), or that high expression of these adhesion molecules is particularly critical during engraftment.

The HSC pool is heterogeneous in reconstitution potential as well as lineage output and is subject to change with age and external signals. In contrast to HSCs from adult bone marrow, fetal liver (FL)-HSCs show a high reconstitution potential in combination with extensive proliferation and a metabolism driven mainly by oxidative phosphorylation. Recent publications have identified molecular (Li et al., 2020), as well as functional heterogeneity (Gao et al., 2022), also within the FL-HSC compartment. Although it is well established that FL-HSCs broadly express MAC-1 (Morrison et al., 1995), it would be interesting investigate in more detail if there are functionally distinct subsets of FL-HSCs displaying differential MAC-1 expression levels, similar to what we have observed for adult stressed HSCs.

To summarize, we demonstrate that differences in cell cycle and transcriptional profile between regenerating MAC-1$^-$ and MAC-1$^+$ HSCs correlate with functional capacity *in vivo*, assessed in secondary and tertiary transplantations, as well as in transplantation after 5FU treatment. Our findings enable the identification and purification of long-term reconstituting HSCs during active regeneration, without the use of genetically modified mouse models or other compromising labeling techniques, and may give new insights into mechanisms regulating maintenance of HSCs and their return to quiescence following regenerative stress.

EXPERIMENTAL PROCEDURES

Resource availability

Corresponding author

For further information, please contact Jonas Larsson (Jonas.Larsson@med.lu.se).

Materials availability

This study did not generate new unique reagents.

Data and code availability

RNA sequencing (RNA-seq) data are available at the NCBI GEO repository with accession number GEO: GSE223648.

Mice

Animals were housed in ventilated racks with free access to autoclaved food and water at the Biomedical Center, Lund University. All experiments were approved by the Lund University Animal Ethical Committee (permit 8042/2020). LY5.1 (B6SJL) and F1 (B6SJL x C57Bl/6) mice were obtained from in-house breeding.

LY5.2 (C57Bl/6NTac) mice were obtained from Taconic. Conventional KO mice B6.129S4-*Itgam*^{tm1Myd}/J (*Itgam*$^{-/-}$) and LY5.2 (C57Bl/6J) were obtained from Jackson. F1 offspring were bred to obtain *Itgam*$^{-/-}$ and *Itgam*$^{+/+}$ littermates used for bone marrow analyses and competitive transplantation. UBC-GFP mice (C57BL/6-Tg(UBC-GFP)30Scha/J) were obtained from Jackson.

Peripheral blood and bone marrow preparation

Peripheral blood from tail vein was collected in Microvette tubes (Sarstedt). Before antibody staining, erythrocytes were lysed with NH_4Cl (STEMCELL Technologies). Bone marrow cells were obtained by crushing femur, tibia, iliac, spine, and sternum using a mortar and pestle, and the cell suspension was filtered through a 40 μm cell strainer. For cKIT enrichment, anti-cKIT magnetic beads were used together with MACS LS columns (Miltenyi Biotec). For lineage depletion, biotinylated antibodies against GR-1, CD3e, B220, TER119 (Biolegend) were used in combination with anti-biotin microbeads (Miltenyi Biotec), followed by separation using MACS LS columns.

Flow cytometry analysis and cell sorting

For transplantation experiments described in Figures 1C and 2D, LSK cells were sorted from cKIT-enriched bone marrow cells stained with CD3e-PECy5 (145-2C11); B220-PECy5 (RA3-6B2); GR1-PECy5 (RB6-8C5); TER119-PECy5 (TER-119); NK1.1-PECy5 (PK136); and SCA1-BV421 (D7), all from BioLegend, and cKIT-APC-eFluor780 (2B8, eBioscience). HSCs (LSK, CD48^{-}, CD150^{+}) were sorted from cKit-enriched bone marrow cells stained with the antibodies above together with CD48-APC (HM48-01, BioLegend), CD150-PECy7 (TC15-12F12.2, BioLegend), and CD11B-fluorescein isothiocyanate (FITC; M1/70, BioLegend). CD45.1-PE (A20, BioLegend) was used to label donor cells in experiment described in Figure 1C and, in combination with CD45.2-BV785 (104, BioLegend), in experiments described in Figures 2D and 4D.

For transplantation experiment described in Figure 4D, HSCs (Lin^{-}, SCA1^{+}, CD48^{-}, CD150^{+}) were sorted from lineage-depleted bone marrow cells stained with the antibodies described above. For transplantations using UBC-GFP mice, the following antibodies were used: CD3e-PECy5; B220-PECy5; GR1-PECy5; TER119-PECy5; NK1.1-PECy5; SCA1-BV421; cKIT-APC-eFluor780; CD48-PE; CD150-PECy7; CD11B-BV785; and EPCR-APC (RCR-16, BioLegend). Cells were sorted on FACS Aria III or FACSymphony (BD).

Whole bone marrow (WBM) staining for myeloerythroid progenitor populations (Pronk et al., 2007) was done using CD3e-PECy5; B220-PECy5; GR1-PECy5; TER119-PECy5; NK1.1-PECy5; SCA1-BV421; cKIT-APC-eFluor780; and CD150-PECy7 as described above, in combination with CD41-FITC (MWReg30, BD Biosciences), CD16/32-APC (93, BioLegend), and CD105-PE (MJ7/18, Santa Cruz). Dead cells were excluded from all BM sorts and analyses using 7-aminoactinomycin D (7AAD).

Peripheral blood was stained using CD45.1-FITC; CD45.2-PE; B220-APC; CD3e-APC; B220-PECy5; CD11B-PECy5; and GR1-PECy5. Blood from mice transplanted with GFP cells were either stained using CD41-PE and TER119-PECy5 before red blood cell lysis or after lysis using B220-APC, CD3e-APC, B220-PECy5, CD11B-PECy5, and GR1-PECy5. In Figure S3C, the following antibodies were used: CD166-PE (eBioALC48, eBioscience), CXCR4-BV421 (L276F12, BioLegend), and CD18-FITC (M18/2, BioLegend). Cells were analyzed on LSRFortessa or LSRFortessa X-20 (BD).

All analysis was performed using FlowJo software (Tree Star).

Transplantations

For serial transplantations described in Figures 1C, 8,000 LSK cells from LY5.1 mice were sorted and transplanted into lethally irradiated (900 cGy) LY5.2 primary recipients together with 2×10^5 WBM from LY5.2 mice. From these primary recipients, 200 LY5.1 HSCs were sorted based on MAC-1 expression and transplanted into LY5.2 secondary recipients together with 2×10^5 WBM from LY5.2 mice. 2×10^6 WBM cells were transplanted to tertiary LY5.2 recipients. For transplantation of SS HSCs, 200 LY5.1 HSCs were sorted based on MAC-1 expression and transplanted into LY5.2 primary recipients together with 2×10^5 WBM from LY5.2 mice. For transplantation of equivalent cell numbers of MAC-1^{low} and MAC-1^{-} HSCs to 200 MAC-1^{+} HSCs (Figure S1D), we sorted the three populations at the same time and subsequently transplanted the same proportion of each collected sample using 200 MAC-1^{+} HSCs as reference.

For transplantations in Figures S1E–S1H, 8,000 LSK cells from UBC-GFP mice were sorted and transplanted to primary LY5.2 recipients together with 2×10^5 WBM from LY5.2 mice. From these primary recipients, 200 ESLAM HSCs were sorted and transplanted to LY5.2 recipients together with 2×10^5 WBM from LY5.2 mice. For transplantations in Figures S1I and S1J, 8,000 LSK cells from UBC-GFP mice were sorted and transplanted to primary LY5.2 recipients together with 2×10^5 WBM from LY5.2 mice. From these primary recipients, we sorted 4 week non-LSK SLAM cells based on MAC-1 expression and transplanted the equivalent dose of each population to that of 200 MAC-1^{+} LSK SLAM HSCs to secondary LY5.2 recipients together with 2×10^5 WBM from LY5.2 mice. For transplantations described in Figure 2D, 4,000 LSK cells from LY5.2 *Itgam* WT or KO mice and 4,000 LY5.1 competitor LSK cells were transplanted together with 2×10^5 WBM F1 support into F1 primary recipients. Equal numbers (either 100 or 300) of LY5.1 and LY5.2 HSCs were sorted from the same primary recipient and transplanted to F1 secondary recipients.

For transplantations described in Figure 4D, 200 HSCs from control and 5FU-treated LY5.1 mice were sorted and transplanted into lethally irradiated (900 cGy) F1 recipients together with 2×10^5 WBM from F1 mice.

Fluorescence microscopy

HSCs were stained as described for Figure 1C but with CD11B conjugated to AF488 instead of FITC and sorted onto Polysine glass slides. Cells were fixed immediately with BD Cytofix/Cytoperm Fixation for 20 min, washed with PBS, and mounted with Fluoromount aqueous mounting medium (Sigma-Aldrich). The slides were analyzed using a Zeiss 780 confocal laser scanning microscope.

qPCR

250 or 500 HSCs were sorted into 8 μL lysis buffer (0.4% NP40, 65 μM dNTP, 2.3 mM DTT, 1.2 mM RNAseOut). cDNA synthesis

followed directly by 10 cycles of targeted preamplification was done using CellsDirect Kit (Invitrogen, #11753100) with pooled gene-specific TaqMan probes (*Actb*: Mm01205647_g; *Itgam*: Mm00434455_m1; *Itgb2*: Mm00434513_m1) (Thermo Fisher Scientific). 4 μL preamplified cDNA was used for the downstream qPCR using Taqman gene expression master mix (Thermo Fisher Scientific, #4369016) together with the individual probes listed above and run on 7900HT Fast Real-Time PCR system (Applied Biosystems). Gene expression levels were normalized to the house-keeping gene *ActB* and adjusted to 1 for the SS MAC-1^{-} samples.

RNA sequencing and analysis

400–600 HSCs/sample were sorted into 10 μL PBS + RNAse inhibitor and snap frozen at −80°C. cDNA synthesis and amplification were done using SMART-Seq v.4 (Takara) and AMPure XP (Beckman Coulter) according to manufacturer instructions. For concentration measurements and size quality controls, Qubit dsDNA HS (Thermo Fisher Scientific) and Bioanalyzer High Sensitivity DNA analysis (Agilent) were used. Library preparation was done using Nextera XT DNA library prep kit (Illumina) according to manufacturer instructions except for library normalization, which was based on Qubit concentration measurements in combination with Bioanalyzer mean library size measurements as follows: molarity (nM) = (ng/μL × 10^6)/(649 × average library size (bp)). Before pooling, each library was diluted to 2 nM. Sequencing was done using NextSeq 500 High output v.2, 150 cycles (Illumina).

Reads were analyzed using the RNA-seq pipeline v.1.4.2 and the STAR alignment in the Nextflow nf-core pipeline (Ewels et al., 2020). Variably expressed genes were found by calculating the variance from a fitted means using DESeq2 normalized data (http://pklab.med.harvard.edu/scw2014/subpop_tutorial.html). PCA was performed using data from the top 1,000 variable genes using the prcomp function in R. Differentially expressed genes were identified using the R package DESeq2 with padj <0.1 and log2FC >1 as thresholds. GSEA was performed by computing overlaps with our dataset and MSigDB gene sets using the GSEA software tool from Broad Institute (Subramanian et al., 2005). Venn diagrams were created using Venny 2.1 (https://bioinfogp.cnb.csic.es/tools/venny/index.html). Overlap in gene expression between two gene sets (RF) were calculated accordingly in combination with normal approximation probability:

RF = x/((n*D)/N), where

x = # of genes in common between groups

n = # of genes in group 1

D = # of genes in group 2

N = # of total genes, in this case 25,000 (estimate of protein coding genes)

Cell-cycle analysis

cKIT enriched (SS and after transplantation) or WBM cells (after 5FU) stained with HSC markers were fixed using BD Cytofix/Cytoperm Fixation and Permeabilization Kit (BD), permeabilized in 0.25% Triton X-100 and stained with anti-KI67 antibody and DAPI. Cells were analyzed on FACS LSRII on low flow rate and an acquisition rate below 1,000 events/s.

Colony assay

50 HSCs were sorted into SFEM (STEMCELL Technologies), transferred to 1.5 mL MethoCult (M3434, STEMCELL Technologies), and cultured for 13 days in a 35 mm culture dish at 37°C, 5% CO_2. Single colonies were transferred to PBS, vortexed, washed, and stained with CD41-PE, TER119-PECy7, GR1-FITC, and CD11B-APC and analyzed on FACS Fortessa. Colony type was based on the following criteria: GM (CD11B > 95%); GEMM (95% > CD11B > 5%, TER119 > 1%, CD41 > 1%); GMMk (95% > CD11B > 5%, TER119 < 1%, CD41 > 1%); and EMk (5% > CD11B, TER119 > 1%, CD41 > 1%).

Statistical analysis

Results are expressed as mean, and error bars represent SD. Statistical significance was determined using two-tailed unpaired Student's t test or one-way ANOVA in combination with Turkey's multiple comparisons test (*p < 0.05, **p < 0.01, ***p < 0.001, ****p < 0.0001). Statistical analysis was performed with GraphPad Prism9 software.

SUPPLEMENTAL INFORMATION

Supplemental information can be found online at https://doi.org/10.1016/j.stemcr.2023.01.014.

AUTHOR CONTRIBUTIONS

A.R. and J.L. planned and designed the experiments with input from E.M., V.S., and K.M. A.R. performed all experiments with assistance from E.M., J.S., and V.S. S.S. performed the bioinformatic analysis with assistance from A.R. A.R. and J.L. wrote the manuscript with input from EM.

ACKNOWLEDGMENTS

We thank Jonas Ungerbäck for technical assistance with the RNA-seq, David Bryder for valuable discussions, and Natsumi Miharada for assistance with animal experiments. This work was funded by grants from the Swedish Research Council and the Swedish Cancer Foundation to J.L.

CONFLICT OF INTERESTS

The authors declare no competing interests.

Received: February 9, 2022
Revised: January 27, 2023
Accepted: January 30, 2023
Published: March 2, 2023

REFERENCES

Bernitz, J.M., Rapp, K., Daniel, M.G., Shcherbinin, D., Yuan, Y., Gomes, A., Waghray, A., Brosh, R., Lachmann, A., Ma'ayan, A., et al. (2020). Memory of divisional history directs the continuous process of primitive hematopoietic lineage commitment. Stem Cell Rep. *14*, 561–574. https://doi.org/10.1016/j.stemcr.2020.03.005.

Bowman, R.L., Busque, L., and Levine, R.L. (2018). Clonal hematopoiesis and evolution to hematopoietic malignancies. Cell Stem Cell *22*, 157–170. https://doi.org/10.1016/j.stem.2018.01.011.

Cao, X., Wu, X., Frassica, D., Yu, B., Pang, L., Xian, L., Wan, M., Lei, W., Armour, M., Tryggestad, E., et al. (2011). Irradiation induces bone injury by damaging bone marrow microenvironment for stem cells. Proc. Natl. Acad. Sci. USA *108*, 1609–1614. https://doi.org/10.1073/pnas.1015350108.

Chitteti, B.R., Kobayashi, M., Cheng, Y., Zhang, H., Poteat, B.A., Broxmeyer, H.E., Pelus, L.M., Hanenberg, H., Zollman, A., Kamocka, M.M., et al. (2014). CD166 regulates human and murine hematopoietic stem cells and the hematopoietic niche. Blood *124*, 519–529. https://doi.org/10.1182/blood-2014-03-565721.

Coxon, A., Rieu, P., Barkalow, F.J., Askari, S., Sharpe, A.H., von Andrian, U.H., Arnaout, M.A., and Mayadas, T.N. (1996). A novel role for the beta 2 integrin CD11b/CD18 in neutrophil apoptosis: a homeostatic mechanism in inflammation. Immunity *5*, 653–666. https://doi.org/10.1016/s1074-7613(00)80278-2.

Ewels, P.A., Peltzer, A., Fillinger, S., Patel, H., Alneberg, J., Wilm, A., Garcia, M.U., Di Tommaso, P., and Nahnsen, S. (2020). The nf-core framework for community-curated bioinformatics pipelines. Nat. Biotechnol. *38*, 276–278. https://doi.org/10.1038/s41587-020-0439-x.

Foudi, A., Jarrier, P., Zhang, Y., Wittner, M., Geay, J.F., Lecluse, Y., Nagasawa, T., Vainchenker, W., and Louache, F. (2006). Reduced retention of radioprotective hematopoietic cells within the bone marrow microenvironment in CXCR4-/- chimeric mice. Blood *107*, 2243–2251. https://doi.org/10.1182/blood-2005-02-0581.

Gao, S., Shi, Q., Zhang, Y., Liang, G., Kang, Z., Huang, B., Ma, D., Wang, L., Jiao, J., Fang, X., et al. (2022). Identification of HSC/MPP expansion units in fetal liver by single-cell spatiotemporal transcriptomics. Cell Res. *32*, 38–53. https://doi.org/10.1038/s41422-021-00540-7.

Jeannet, R., Cai, Q., Liu, H., Vu, H., and Kuo, Y.H. (2013). Alcam regulates long-term hematopoietic stem cell engraftment and self-renewal. Stem Cell. *31*, 560–571. https://doi.org/10.1002/stem.1309.

Kiel, M.J., Yilmaz, O.H., Iwashita, T., Yilmaz, O.H., Terhorst, C., and Morrison, S.J. (2005). SLAM family receptors distinguish hematopoietic stem and progenitor cells and reveal endothelial niches for stem cells. Cell *121*, 1109–1121. https://doi.org/10.1016/j.cell.2005.05.026.

Komorowska, K., Doyle, A., Wahlestedt, M., Subramaniam, A., Debnath, S., Chen, J., Soneji, S., Van Handel, B., Mikkola, H.K.A., Miharada, K., et al. (2017). Hepatic leukemia factor maintains quiescence of hematopoietic stem cells and protects the stem cell pool during regeneration. Cell Rep. *21*, 3514–3523. https://doi.org/10.1016/j.celrep.2017.11.084.

Li, Y., Kong, W., Yang, W., Patel, R.M., Casey, E.B., Okeyo-Owuor, T., White, J.M., Porter, S.N., Morris, S.A., and Magee, J.A. (2020). Single-cell analysis of neonatal HSC ontogeny reveals gradual and uncoordinated transcriptional reprogramming that begins before birth. Cell Stem Cell *27*, 732–747.e7. https://doi.org/10.1016/j.stem.2020.08.001.

Mei, Y., Han, X., Liu, Y., Yang, J., Sumagin, R., and Ji, P. (2020). Diaphanous-related formin mDia2 regulates beta2 integrins to control hematopoietic stem and progenitor cell engraftment. Nat. Commun. *11*, 3172. https://doi.org/10.1038/s41467-020-16911-4.

Morrison, S.J., Hemmati, H.D., Wandycz, A.M., and Weissman, I.L. (1995). The purification and characterization of fetal liver hematopoietic stem cells. Proc. Natl. Acad. Sci. USA *92*, 10302–10306.

Nie, Y., Han, Y.C., and Zou, Y.R. (2008). CXCR4 is required for the quiescence of primitive hematopoietic cells. J. Exp. Med. *205*, 777–783. https://doi.org/10.1084/jem.20072513.

Pietras, E.M., Lakshminarasimhan, R., Techner, J.M., Fong, S., Flach, J., Binnewies, M., and Passegué, E. (2014). Re-entry into quiescence protects hematopoietic stem cells from the killing effect of chronic exposure to type I interferons. J. Exp. Med. *211*, 245–262. https://doi.org/10.1084/jem.20131043.

Prashad, S.L., Calvanese, V., Yao, C.Y., Kaiser, J., Wang, Y., Sasidharan, R., Crooks, G., Magnusson, M., and Mikkola, H.K.A. (2015). GPI-80 defines self-renewal ability in hematopoietic stem cells during human development. Cell Stem Cell *16*, 80–87. https://doi.org/10.1016/j.stem.2014.10.020.

Pronk, C.J.H., Rossi, D.J., Månsson, R., Attema, J.L., Norddahl, G.L., Chan, C.K.F., Sigvardsson, M., Weissman, I.L., and Bryder, D. (2007). Elucidation of the phenotypic, functional, and molecular topography of a myeloerythroid progenitor cell hierarchy. Cell Stem Cell *1*, 428–442. https://doi.org/10.1016/j.stem.2007.07.005.

Qiu, J., Papatsenko, D., Niu, X., Schaniel, C., and Moore, K. (2014). Divisional history and hematopoietic stem cell function during homeostasis. Stem Cell Rep. *2*, 473–490. https://doi.org/10.1016/j.stemcr.2014.01.016.

Randall, T.D., and Weissman, I.L. (1997). Phenotypic and functional changes induced at the clonal level in hematopoietic stem cells after 5-fluorouracil treatment. Blood *89*, 3596–3606.

Rodriguez-Fraticelli, A.E., Weinreb, C., Wang, S.W., Migueles, R.P., Jankovic, M., Usart, M., Klein, A.M., Lowell, S., and Camargo, F.D. (2020). Single-cell lineage tracing unveils a role for TCF15 in haematopoiesis. Nature *583*, 585–589. https://doi.org/10.1038/s41586-020-2503-6.

Scharffetter-Kochanek, K., Lu, H., Norman, K., van Nood, N., Munoz, F., Grabbe, S., McArthur, M., Lorenzo, I., Kaplan, S., Ley, K., et al. (1998). Spontaneous skin ulceration and defective T cell function in CD18 null mice. J. Exp. Med. *188*, 119–131. https://doi.org/10.1084/jem.188.1.119.

Subramanian, A., Tamayo, P., Mootha, V.K., Mukherjee, S., Ebert, B.L., Gillette, M.A., Paulovich, A., Pomeroy, S.L., Golub, T.R., Lander, E.S., and Mesirov, J.P. (2005). Gene set enrichment analysis: a knowledge-based approach for interpreting genome-wide expression profiles. Proc. Natl. Acad. Sci. USA *102*, 15545–15550. https://doi.org/10.1073/pnas.0506580102.

Takizawa, H., Regoes, R.R., Boddupalli, C.S., Bonhoeffer, S., and Manz, M.G. (2011). Dynamic variation in cycling of hematopoietic stem cells in steady state and inflammation. J. Exp. Med. *208*, 273–284. https://doi.org/10.1084/jem.20101643.

Vitali, C., Bassani, C., Chiodoni, C., Fellini, E., Guarnotta, C., Miotti, S., Sangaletti, S., Fuligni, F., De Cecco, L., Piccaluga, P.P., et al. (2015). SOCS2 controls proliferation and stemness of hematopoietic cells under stress conditions and its deregulation marks unfavorable acute leukemias. Cancer Res. *75*, 2387–2399. https://doi.org/10.1158/0008-5472.Can-14-3625.

Walter, D., Lier, A., Geiselhart, A., Thalheimer, F.B., Huntscha, S., Sobotta, M.C., Moehrle, B., Brocks, D., Bayindir, I., Kaschutnig, P., et al. (2015). Exit from dormancy provokes DNA-damage-induced attrition in haematopoietic stem cells. Nature *520*, 549–552. https://doi.org/10.1038/nature14131.

Wang, J., Liu, Z., Zhang, S., Wang, X., Bai, H., Xie, M., Dong, F., and Ema, H. (2019). Lineage marker expression on mouse hematopoietic stem cells. Exp. Hematol. *76*, 13–23.e2. https://doi.org/10.1016/j.exphem.2019.07.001.

Wang, W., Yu, S., Zimmerman, G., Wang, Y., Myers, J., Yu, V.W.C., Huang, D., Huang, X., Shim, J., Huang, Y., et al. (2015). Notch receptor-ligand engagement maintains hematopoietic stem cell quiescence and niche retention. Stem Cell. *33*, 2280–2293. https://doi.org/10.1002/stem.2031.

Wilson, A., Laurenti, E., Oser, G., van der Wath, R.C., Blanco-Bose, W., Jaworski, M., Offner, S., Dunant, C.F., Eshkind, L., Bockamp, E., et al. (2008). Hematopoietic stem cells reversibly switch from dormancy to self-renewal during homeostasis and repair. Cell *135*, 1118–1129. https://doi.org/10.1016/j.cell.2008.10.048.

Stem Cell Reports, Volume *18*

Supplemental Information

MAC-1 marks a quiescent and functionally superior HSC subset during regeneration

Anna Rydström, Els Mansell, Valgardur Sigurdsson, Julia Sjöberg, Shamit Soneji, Kenichi Miharada, and Jonas Larsson

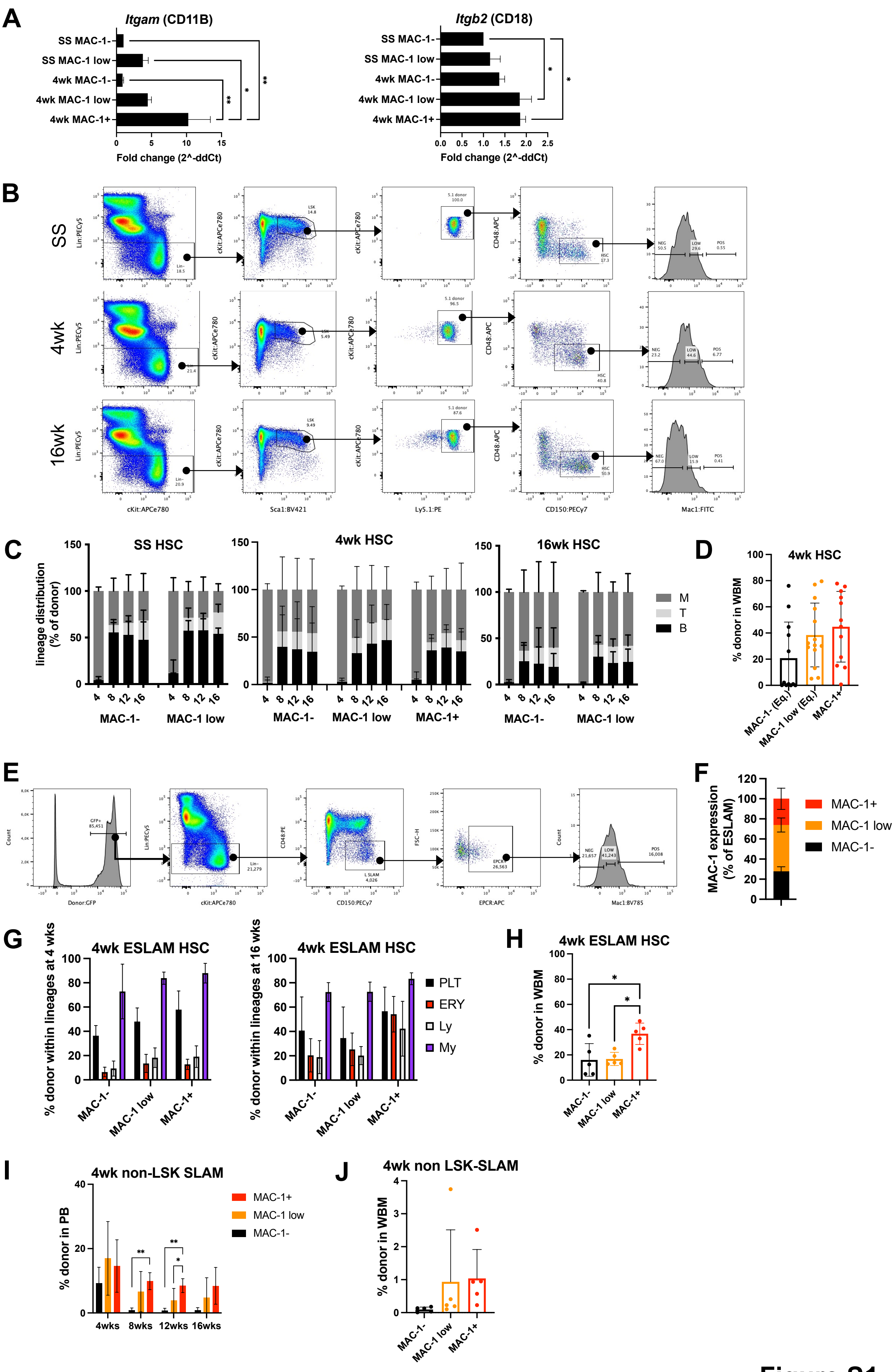
A
Itgam (CD11B)
SS MAC-1-
SS MAC-1 low
4wk MAC-1-
4wk MAC-1 low
4wk MAC-1+
Fold change (2^-ddCt)
Itgb2 (CD18)
Fold change (2^-ddCt)
B
SS
4wk
16wk
Lin:PECy5
cKit:APCe780
Sca1:BV421
Ly5.1:PE
CD48:APC
CD150:PECy7
Mac1:FITC
C
SS HSC
4wk HSC
16wk HSC
lineage distribution (% of donor)
MAC-1-
MAC-1 low
MAC-1+
M
T
B
D
4wk HSC
% donor in WBM
MAC-1- (Eq.)
MAC-1 low (Eq.)
MAC-1+
E
Donor:GFP
cKit:APCe780
CD150:PECy7
EPCR:APC
Mac1:BV785
F
MAC-1 expression (% of ESLAM)
MAC-1+
MAC-1 low
MAC-1-
G
4wk ESLAM HSC
% donor within lineages at 4 wks
% donor within lineages at 16 wks
PLT
ERY
Ly
My
H
4wk ESLAM HSC
% donor in WBM
I
4wk non-LSK SLAM
% donor in PB
4wks 8wks 12wks 16wks
J
4wk non LSK-SLAM
% donor in WBM

Figure S1

Figure S1. BM and PB analysis after transplantation. Related to Figure 1.
(A) Gene expression analysis by qPCR of *Itgam* and *Itgb2* in SS and 4wk HSCs sorted based on MAC-1 expression. Data are represented as mean ± SD. (n=3 mice). Statistical significance was determined using one-way ANOVA in combination with Turkey's multiple comparisons test (*p<0.05), (**p<0.01). (B) Representative dot plots of FACS of LSK SLAM HSCs from SS mice and from mice 4 or 16 weeks after transplantation. (C) Lineage output over time in donor PB shown in Figure 1D. Data are represented as mean ± SD. (D) Engraftment in BM 16 weeks after transplantation of equivalent cell numbers to 200 MAC-1$^+$ HSCs. The MAC-1$^+$ bar is the same as in (Figure 1E). Data pooled from 2 experiments (n=12-14 mice). Data are represented as mean ± SD. (E) Representative dot plots of FACS of ESLAM HSCs from mice 4 weeks after transplantation. (F) Proportion of HSCs in (E) being MAC-1$^-$, MAC-1^{low} and MAC-1$^+$. (G) Engraftment of 4wk ESLAM HSCs at 4 and 16 weeks in PB and at 16 weeks in BM (H). (n=5 mice for F-H). Data are represented as mean ± SD. Statistical significance was determined using one-way ANOVA in combination with Turkey's multiple comparisons test (*p<0.05), (**p<0.01). (I) Engraftment over time in donor PB and BM (J) after transplantation of 4wk non-LSK SLAM cells. (n=5 mice). Data are represented as mean ± SD. Statistical significance was determined using one-way ANOVA in combination with Turkey's multiple comparisons test (*p<0.05), (**p<0.01).

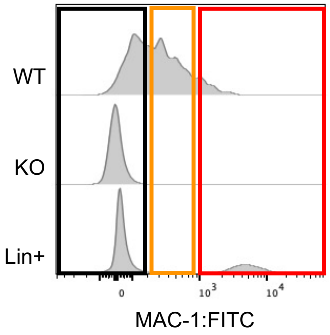

Figure S2

Figure S2. MAC-1 expression in WT and *Itgam* KO HSCs. Related to Figure 2.
Histograms of HSCs (LSK-SLAM) from WT and KO mice 4 weeks after transplantation. Lineage positive (Lin^+) cells are displayed as reference.

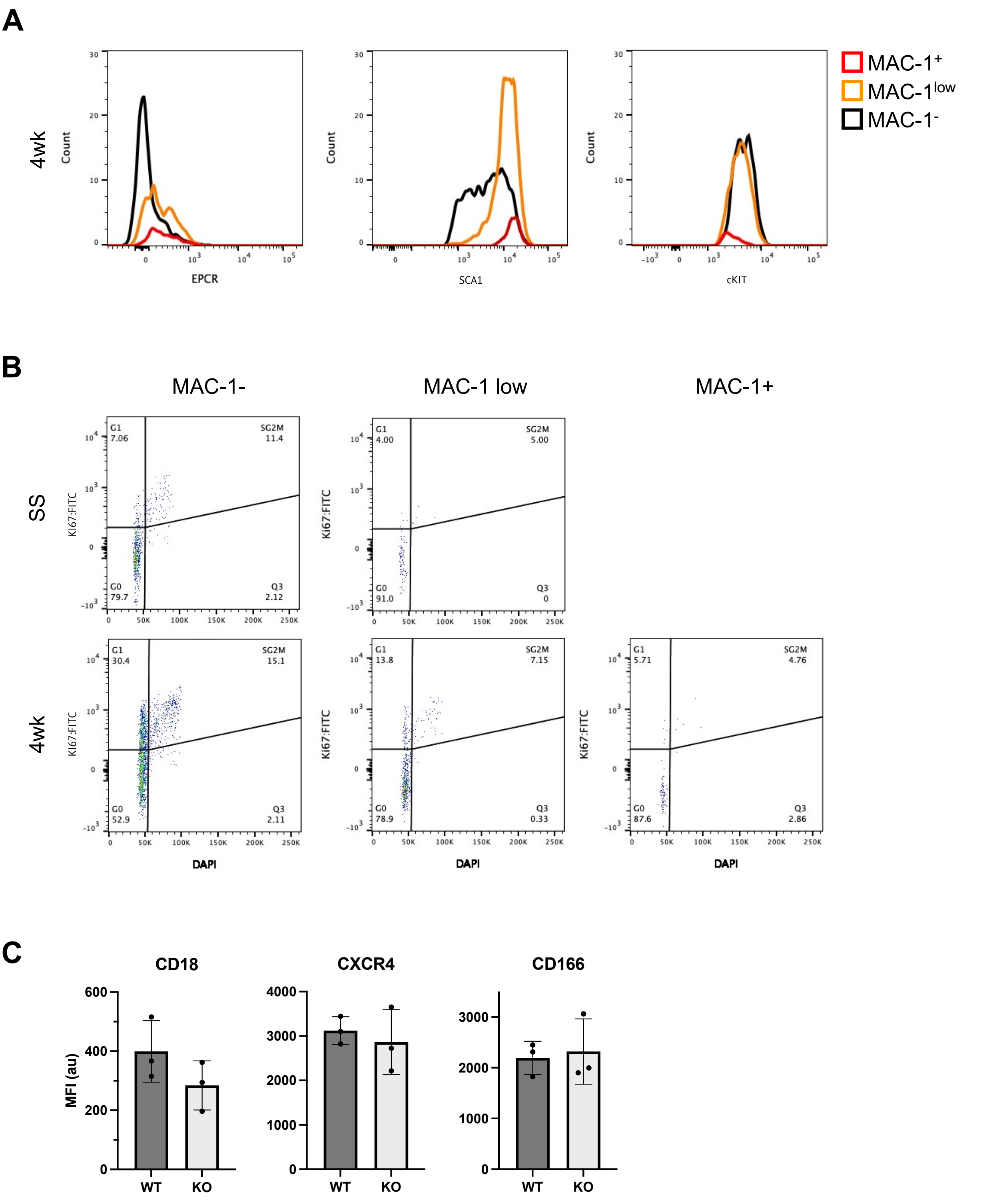
A
4wk
Count
EPCR
SCA1
cKIT
MAC-1+
MAC-1low
MAC-1-
B
MAC-1-
MAC-1 low
MAC-1+
SS
4wk
Ki67:FITC
DAPI
G1 7.06
SG2M 11.4
G0 79.7
Q3 2.12
G1 4.00
SG2M 5.00
G0 91.0
Q3 0
G1 30.4
SG2M 15.1
G0 52.9
Q3 2.11
G1 13.8
SG2M 7.15
G0 78.9
Q3 0.33
G1 5.71
SG2M 4.76
G0 87.6
Q3 2.86
C
CD18
CXCR4
CD166
MFI (au)
WT
KO

Figure S3

Figure S3. Protein expression and cell cycle analysis on HSCs. Related to Figure 3.
(A) Representative histograms of cell surface protein expression of EPCR, SCA1 and cKIT on 4wk MAC-1 LSK SLAM HSC subpopulations. (B) Representative dot plots of flow cytometry analysis of HSCs from SS mice and from mice 4 weeks after transplantation based on their MAC-1 expression. (C) Protein expression of adhesion molecules CD18, CXCR4 and CD166 on HSCs from *Itgam* KO and WT littermates. (n=3 mice). Data are represented as mean ± SD.
